# HDAC2 enhances esophageal squamous cell carcinoma development through down-regulating microRNA-503-5p and promoting CXCL10

**DOI:** 10.1186/s13148-021-01068-8

**Published:** 2021-04-29

**Authors:** Jindong Li, Chengyan Jin, Lihua Sun, Bin Wang, Peiyan Hua, Yan Zhang

**Affiliations:** 1 Department of Thoracic Surgery, The Second Hospital of Jilin University, NO. 218 Ziqiang Street, Changchun, 130041 Jilin China; 2 Department of Anesthesiology, The Second Hospital of Jilin University, Changchun, 130041 Jilin China

**Keywords:** Esophageal squamous cell carcinoma, Histone deacetylase 2, MicroRNA-503-5p, C-X-C motif chemokine 10, Apoptosis, Tumorigenesis

## Abstract

**Objective:**

Although esophageal squamous cell carcinoma (ESCC)-oriented mechanism has been widely explored, the integrated action of histone deacetylase 2 (HDAC2), microRNA (miR)-503-5p and C-X-C motif chemokine 10 (CXCL10) in ESCC has not been thoroughly explored. Thus, we performed the research to study the role of HDAC2/miR-503-5p/CXCL10 axis in ESCC.

**Methods:**

ESCC tissues and mucosal tissues (5 cm from cancer tissues) were collected, in which HDAC2, miR-503-5p and CXCL10 expression levels were tested. The mechanism of HDAC2, miR-503-5p and CXCL10 was interpreted. The viability, colony formation ability, apoptosis, invasion and migration abilities of ESCC cells were tested after HDAC2, miR-503-5p or CXCL10 expression was altered. Tumorigenesis in mice was observed to further verify the in vitro effects of HDAC2 and miR-503-5p.

**Results:**

HDAC2 and CXCL10 were up-regulated while miR-503-5p was down-regulated in ESCC. HDAC2 bound to miR-503-5p and miR-503-5p targeted CXCL10. Silencing HDAC2 or restoring miR-503-5p depressed viability, colony-forming, invasion and migration abilities and enhanced apoptosis of ESCC cells in vitro, as well as suppressed ESCC tumorigenesis in vivo. Inhibition of miR-503-5p or elevation of CXCL10 negated HDAC2 knockout-induced effects on ESCC cells.

**Conclusion:**

This work elucidates that HDAC2 knockdown retards the process of ESCC by elevating miR-503-5p and inhibiting CXCL10 expression, which may provide a guidance for ESCC management.

**Supplementary Information:**

The online version contains supplementary material available at 10.1186/s13148-021-01068-8.

## Introduction

Characterized by invasive and metastatic properties with dismal recurrence, esophageal squamous cell carcinoma (ESCC) is the overwhelmed esophageal cancer (EC) in East Asia [1]. Blamed on no identified clinical symptoms in the early stages, most ESCC patients are diagnosed in a middle or advanced stage [2]. As estimated, the prognosis of ESCC is beneath expectancy, with only 15% 5-year survival rate [3]. The well-established therapies for ESCC comprise of surgery, chemoradiation and radiotherapy [4]. Specifically, radiotherapy with concurrent chemoradiotherapy confers an improved survival for non-surgical ESCC patients, and consolidation chemotherapy after concurrent chemoradiotherapy attenuates progression-free survival [5]. Despite technique improvements in ESCC management, the mystery about ESSC initiation and development needs to be explored thoroughly.

Histone deacetylases (HDACs) inhibitors are anti-tumorigenic, as evidenced by their potentials in arresting cell cycle progression, inhibiting differentiation/angiogenesis and inducing apoptosis by modification of cellular proteins [6]. Of the component of HDACs, HDAC2 takes part in the mediation of ESCC cell proliferation, cell cycle arrest and apoptosis [7]. Actually, it has been demonstrated that inhibited HDAC2 could enhance apoptosis and repress proliferation of ESCC cells [8]. MicroRNAs (miRNAs) are vital in the biological behaviors of EC cells, and they are often connected with multi-drug resistance of esophageal cancer [9]. It is previously evidenced that miR-503 takes part in the medication of malignant phenotype of ESCC, and it enables itself to disturb ESCC cells to proliferate, invade and migrate via autophagy activation [10]. Moreover, up-regulation of miR-503 represses ESCC cell proliferating, invasive and migrating capacities [11]. In ESCC, the chemokine, C-X-C motif chemokine 10 (CXCL10, also known as IP-10) has been elucidated to function as an anti-tumorigenic actor [12]. Additionally, up-regulated CXCL10 is a promising index for the prognosis of ESCC patients [13]. As manifested by a former study, enhanced histone acetylation is obvious in the CXCL10 promoter consistent with CXCL10 activation [14]. Intriguingly, CXCL10 is confirmed to be a target for miR-503 [15]. Jointly, how the combination of HDAC2, miR-503-5p and CXCL10 functions in the progression of ESCC is indefinable. Enlightened by that, this work was programmed to explore the role of HDAC2/miR-503-5p/CXCL10 axis in ESCC.

## Materials and methods

### Ethics statement

All animal experiments were conducted in compliance with the Guidelines for the Care and Use of Laboratory Animals of the National Institutes of Health, and were approved by the Animal Care and Use Committee of The Second Hospital of Jilin University.

### Experimental subjects

Inpatients who underwent endoscopic census and histological examination in The Second Hospital of Jilin University from 2012 to 2014 were enrolled, and they underwent surgery and were diagnosed with EC. Fresh EC tissues (*n* = 121) and mucosal tissues (*n* = 121; more than 5 cm from EC tissues) were collected. None of patients had accepted treatment before surgery. EC patients included 84 males and 37 females (22.4–76.2 years old). There were 45 cases with lymph node metastasis while 76 cases without; 98 cases with highly and moderately differentiated tumors while 23 cases with poorly differentiated tumors. Inclusion criteria: patients were diagnosed with EC by tumor pathology and genetics; patients had no history of other tumors; patients had no chemotherapy or radiotherapy before surgery. Exclusion criteria: patients with severe organ dysfunction such as heart, liver and kidney; patients with a history of autoimmune disease; patients with active chronic or acute infectious diseases. According to the 7th edition of tumor node metastasis (TNM) staging [16], 63 cases were in stages I–II, and 58 cases in stage III.

### Cell culture

Human ESCC cell lines (KYSE150, KYSE410, KYSE520, Ec109 and KYSE30) and human normal esophageal epithelial cells (HEEC) (all from Shanghai Institutes for Biological Sciences, Chinese Academy of Sciences, Shanghai, China) were cultured in Dulbecco’s modified Eagle medium (DMEM) supplemented with 10% fetal bovine serum (FBS, Hyclone, Logan, UT, USA), 100 U/mL penicillin and 100 μg/mL Amycin (both from Sigma-Aldrich, St. Louis, MO, USA). The two cancer cell lines with higher HDAC2 expression were screened by reverse transcription quantitative polymerase chain reaction (RT-qPCR) for subsequent experiments.

### Cell transfection

The lentivirus targeting human HDAC2 (sh-HDAC2, GenePharma, Shanghai, China), and its corresponding non-targeting negative control (sh-NC) were transfected into ESCC cells. Before transfection, ESCC cells were placed in 3 μg/mL puromycin for 72 h, with the efficiency verified by the percentage of green fluorescent protein-positive cells. miR-503-5p mimic, miR-503-5p inhibitor, miR-503-5p NC and pcDNA-CXCL10 (all from Ribobio, Guangzhou, China) were transfected into ESCC cells by Lipofectamine 2000 (Invitrogen, CA, USA) [17].

TSA (Sigma-Aldrich) was dissolved in dimethyl sulfoxide (DMSO; Sigma-Aldrich) at a concentration of 1 μM [18]. Control cells were treated with DMSO.

### 3-(4, 5-dimethylthiazol-2-yl)-2, 5-diphenyltetrazolium bromide (MTT) assay

Seeded in 96-well plates at 1000 cells/well, ESCC cells were cultured for 24, 48 and 72 h, respectively, and then supplemented with 20 μL MTT solution. Then, ESCC cells were added with 200 μL DMSO and microscopically observed. Finally, optical density (OD, 450 nm) values were recorded on a microplate reader.

### Colony formation assay

ESCC cells (500 cells/well) were seeded on the 6-well plates (Corning, NY, USA), cultured for 2 week and fixed by 4% paraformaldehyde. Followed by that, ESCC cells were stained with 0.1% crystal violet solution. The stained colonies were counted and photographed, and colony formation rate was calculated.

### Flow cytometry

Cells (1 × 106 cells/well) were seeded in a 6-well plate and incubated for 24 h. Using an Annexin V/PI kit (KeyGEN, Nanjing, China), the percentage of apoptotic cells was checked on a FACScalibur flow cytometer (BD Biosciences, San Jose, CA, USA).

### Transwell assay

At 48 h post-transfection, ESCC cells were starved in serum-free medium for 24 h and adjusted to 3 × 10^4^ cells/mL with serum-free medium. Cells (1 × 10^5^) combined with DMEM (200 μL) were spread into the upper chamber coated with Matrigel (1:8, Shanghai Yeasen Biological Technology Co., Ltd., Shanghai, China) while 20% FBS-DMEM (600 μL) into the lower chamber. After 24 h, the invasive cells were fixed with 4% polyoxymethylene and stained with 0.5% crystal violet solution. The infiltrating cells were observed in 5 random fields of view under an inverted microscope (XDS-800D, Caikon Optical Instrument Co., Ltd., Shanghai, China). The upper chamber was not coated with Matrigel in cell migration experiment [19].

### RT-qPCR

Total RNA was isolated from tissues or cells by Trizol (Invitrogen) or GenElute Total RNA Purification Kit (Sigma-Aldrich). Reverse transcription was launched with PrimeScript RT master mix (Takara, Beijing, China) in an S1000 thermal cycler (Bio-Rad, Hercules, CA, USA). Real-time PCR was conducted in a CFX96 real-time system (Bio-Rad) using KAPA SYBR FAST qPCR Master Mix kit (Kapa Biosystem, Wilmington, MA, USA). The sample was subjected to RT-qPCR in a real-time fluorescent quantitative PCR instrument (ABI7500, ABI, CA, USA). All primers are listed in Table 1. Glyceraldehyde-3-phosphate dehydrogenase (GAPDH) or U6 conferred an endogenous reference, and the 2^−ΔΔCt^ [20] method was indicated to calculate gene expression.

**Table 1:** Primer sequences

Gene	Primer sequences
HDAC2	Forward: 5′-CGTGTAATGACGGTATCATTCC-3′
	Reverse: 5′-ACCAGATAATGAGTCTGCACC-3′
miR-503-5p	Forward: 5′-CCTATTTCCCATGATTCCTTCATA-3′
	Reverse: 5′-GTAATACGGTTATCCACGCG-3′
CXCL10	Forward: 5′-TATTCCTGCAAGCCAATTTTGTC-3′
	Reverse: 5′-TCTTGATGGCCTTCGATTCTG-3′
U6	Forward: 5′-CTCGCTTCGGCAGCACA-3′
	Reverse: 5′-AACGCTTCACGAATTTGCGT-3′
GAPDH	Forward: 5′-ACCACCATGGAGAAGGCTGG-3′
	Reverse: 5′-CTCAGTGTAGCCCAGGATGC-3′
E-cadherin	Forward: 5′-TGCACCAACCCTCATGAGTG-3′
	Reverse: 5′-GTCAGTATCAGCCGCTTTCAG-3′
Vimentin	Forward: 5′-GAGAACTTTGCCGTTGAAGC-3′
	Reverse: 5′-TCCAGCAGCTTCCTGTAGGT-3′
PCNA	Forward:5′-GCCATATTGGAGATGCTGT-3′
	Reverse: 5′-TGAGTGTCACCGTTGAAGA-3′

HDAC2, histone deacetylase-2; miR-503-5p, microRNA-503-5p; CXCL10, C-X-C motif chemokine 10; GAPDH, glyceraldehyde-3-phosphate dehydrogenase; E-cadherin, epithelial cadherin; PCNA, proliferating cell nuclear antigen

### Western blot assay

Tissues and cells were lysed with radio-immunoprecipitation assay buffer (Cell Signaling Technology, MA, USA) containing protease inhibitors. Protein concentration was tested by bicinchoninic acid protein analysis kit (Pierce, Rockford, IL, USA). Separated by sodium dodecyl sulfate polyacrylamide gel electrophoresis, protein (30 μg) was transferred to a polyvinylidene fluoride membrane, followed by blockade in 5% skim milk. The protein membrane was probed with primary antibodies HDAC2 (ab32117, 1:1000, Abcam), CXCL10 (ab137018, 1:1000, Abcam) and re-probed with HRP-conjugated goat anti-rabbit immunoglobulin G (IgG) antibody (ab6721, Abcam). With GAPDH (ab8245, 1:1000, Abcam) as an internal control, protein bands were detected by SupreSignal ECL kit (Pierce).

### Dual-luciferase reporter gene assay

Luciferase plasmids (GenePharma) containing wild-type (pmirGLO-CXCL10-WT) or mutant (pmirGLO-CXCL10-MUT) CXCL10 binding sites targeting miR-503-5p were prepared. Lipofectamine 2000 (Thermo Fisher Scientific, MA, USA) was adopted to co-transfect pmirGLO-CXCL10-WT or pmirGLO-CXCL10-MUT with miR-503-5p mimic or miR-NC (Invitrogen) into KYSE30 and Ec109 cells. Luciferase activity was measured by a fluorometer (PerkinElmer Life Sciences, Boston, MA, USA) and dual-luciferase reporter gene detection system (Promega) [21]. The fluorescence intensity was detected with Glomax20/20 Luminometer (E5311, Promega).

### Chromatin immunoprecipitation (ChIP) assay

Cells were lysed by a solution containing 50 mM Tris–HCl, pH 8.1, 1% sodium dodecyl sulfate, 10 mM ethylene diamine tetraacetic acid and complete protease inhibitor cocktail (Roche, Basel, Switzerland). Next, cells were sonified to harvest 200 1000 bp DNA fragments. ChIP was implemented on a ChIP Assay Kit (Millipore, Bedford, MA, USA). Anti-HDAC2 (3F3, sc-81599) and normal mouse IgG (sc-2025) were provided by Santa Cruz (Santa Cruz, CA, USA) [22].

### Tumor xenografts in nude mice

Nude mice (*n* = 10), aged 6 weeks, were supplied by SLAC Laboratory Animal Co., Ltd. (Shanghai, China) and randomly distributed into 2 groups. Ec109 cells transfected with sh-HDAC2 or sh-NC in serum-free suspension (1 × 10^7^ cells/mL) were subcutaneously injected into mice (0.2 mL). When tumors grew to 0.2–1 cm^3^, tumor volume was calculated by 1/2 × Length^2^ × Width (mm). The average tumor volume was measured 3 times every 7 days. At the end of the experiment (day 35), the mice were euthanized to excise tumors. The tumors were fixed, photographed and preserved for hematoxylin–eosin (HE) staining and immunohistochemistry.

### HE staining

Paraffin-embedded xenografted tumors were stained with HE staining solution and analyzed blindly by a pathologist [23].

### Statistical analysis

SPSS 22.0 statistical software (IBM, NY, USA) was used in data analysis. Data were expressed as mean ± standard deviation, representing at least three independent experiments. Discrepancies in groups were tested by Student’s t test or one-way analysis of variance (ANOVA). Chi-square test was adopted to analyze correlation between HDAC2 expression and clinical parameters. Kaplan–Meier method was utilized to survival curve drawing while log-rank test to statistical comparison. Pearson correlation analysis was applied to analyze correlation. At *P* < 0.05, the discrepancy was of statistical significance.

## Results

### HDAC2 is up-regulated in ESCC that is correlated with inferior prognosis

**Fig. 1:**
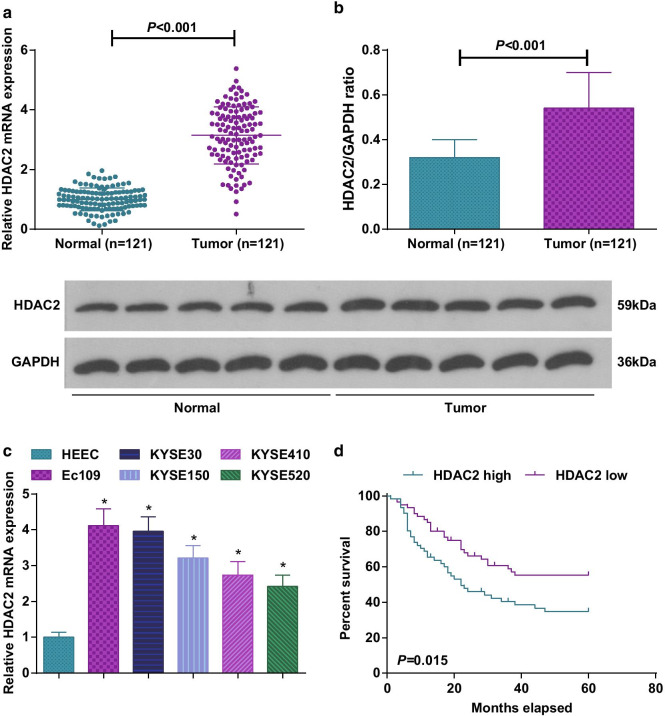
HDAC2 is up-regulated in ESCC tissues and cells, and connected with inferior prognosis. **a** RT-qPCR to detect HDAC2 mRNA expression in ESCC cancer and normal tissues; **b** Western blot to detect HDAC2 protein expression in ESCC cancer and normal tissues; **c** RT-qPCR to detect HDAC2 mRNA expression in ESCC cell lines and HEEC; **d** Log-rank test to analyze survival prognosis of ESCC patients with high and low HDAC2 expression levels; for all quantitative results, the data were expressed as mean ± standard deviation of three independent experiments. Discrepancies between two groups were assessed by Student’s *t* test while those among multiple groups by one-way ANOVA; * *P* < 0.05 compared with HEEC cells

HDAC inhibitors can treat SCC [24], and HDAC2 immunoreactivity is enhanced in ESCC tissues [8]. For clarification of HDAC2-pivoted mechanism in ESCC, we tested HDAC2 expression in 121 cases of ESCC tumor and mucosal tissues, as well as in ESCC cell lines and HEEC by RT-qPCR and Western blot assay. The results manifested that HDAC2 expression was elevated in ESCC cancer tissues (Fig. 1a, b) and cells (Fig. 1c). For follow-up cell experiments, we selected cell lines with higher HDAC2 expression levels (Ec109 and KYSE30).

The correlation between HDAC2 expression and clinicopathological traits of 121 ESCC patients was analyzed. Based on HDAC2 median expression, ESCC patients were allocated into HDAC2 high group (*n* = 61) and HDAC2 low group (*n* = 60). We noticed that HDAC2 expression was correlated with tumor diameter, degree of differentiation and TNM stage (Table 2). Furthermore, we analyzed the prognostic value of HDAC2 expression in ESCC using log-rank test and Kaplan–Meier method and observed that the survival prognosis of ESCC patients with HDAC2 high expression was inferior to that with HDAC2 low expression (Fig. 1d). Collectively, up-regulated HDAC2 in ESCC was correlated with inferior prognosis.

**Table 2:** Relationship between HDAC2 expression and clinicopathological traits of ESCC patients

Clinicopathological data	*n*	HDAC2 expression		*P*	*x*^*2*^
		Low expression group (*n* = 60)	High expression group (*n* = 61)		
*Age (years)*					
≥ 59	49	26	23	0.528	0.4
< 59	72	34	38		
*Gender*					
Male	84	39	45	0.295	1.096
Female	37	21	16		
*Tumor diameter*					
< 5 cm	92	50	42	0.062	3.481
≥ 5 cm	29	10	19		
*Lymph node metastasis*					
Yes	45	18	27	0.105	2.634
No	76	42	34		
*Tumor node metastasis stage*					
I–II	63	38	25	0.014	6.054
III	58	22	36		
*Differentiation degree*					
Well or moderate	98	45	53	0.096	2.775
Poor	23	15	8		

The data in this table are enumeration data, using Chi-square test. HDAC2, histone deacetylase-2; ESCC, esophageal squamous cell carcinoma

### Silencing HDAC2 depresses ESCC cell viability and colony-forming ability and enhances apoptosis

**Fig. 2:**
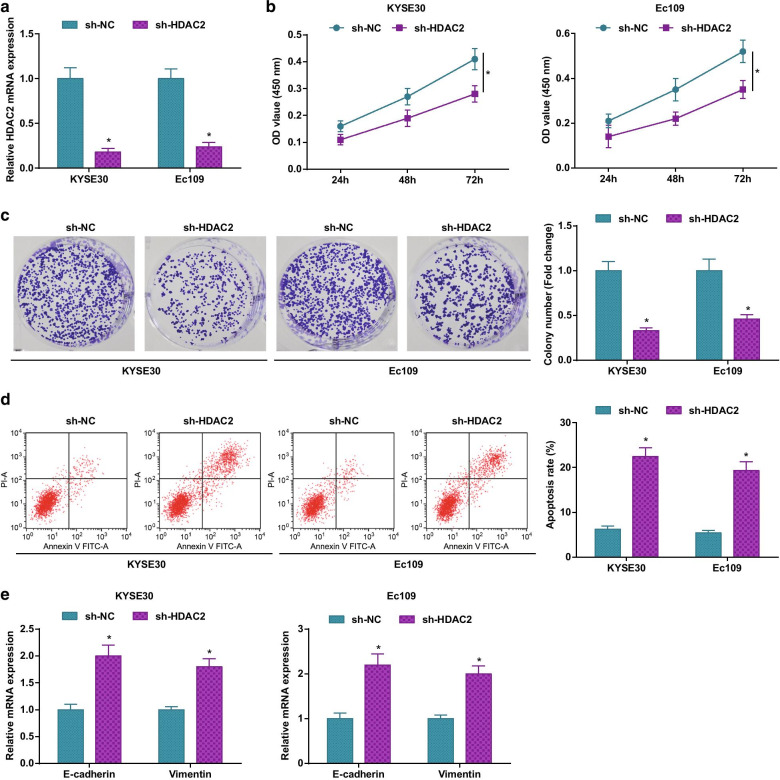
Silencing HDAC2 depresses ESCC cell viability and colony-forming ability, and enhances apoptosis. **a** RT-qPCR to detect HDAC2 mRNA expression in ESCC cells; **b** MTT assay to detect ESCC cell viability; **c** colony formation assay to detect ESCC cell colony-forming abilities; **d** flow cytometry to detect ESCC cell apoptosis rate; **e** RT-qPCR to detect E-cadherin and Vimentin protein expression in ESCC cells; for all quantitative results, the data were expressed as mean ± standard deviation of three independent experiments. Discrepancies among multiple groups were assessed by one-way ANOVA; * *P* < 0.05 compared with the sh-NC group

With the purpose to disclose the relevant functions of dysregulated HDAC2 in ESCC, we further analyzed the biological functions of ESCC cells after interference with HDAC2. We utilized the lentivirus (sh-HDAC2) stably expressing HDAC2 to transfect ESCC cells and verified through RT-qPCR that HDAC2 expression was effectively knocked down in KYSE30 and Ec109 cells (Fig. 2a). Moreover, we found in MTT assay, colony formation assay, flow cytometry and Western blot that KYSE30 and Ec109 cells with down-regulated HDAC2 showed impaired cell viability and colony-forming ability, enhanced apoptosis, elevated E-cadherin expression and reduced Vimentin expression (Fig. 2b–e).

### Silencing HDAC2 limits ESCC cell invasive and migrating properties and tumorigenesis in mice

**Fig. 3:**
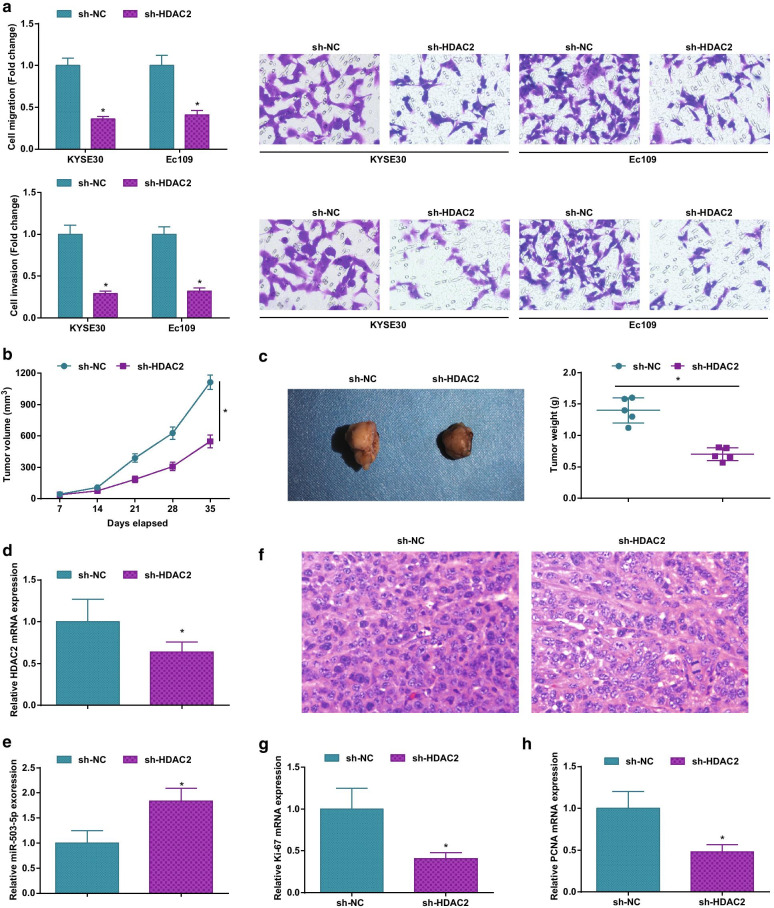
Silencing HDAC2 disturbs ESCC cell invasive and migrating properties and tumorigenesis in mice. **a** Transwell assay to detect ESCC cell migration and invasion abilities; **b** Xenografted tumor volume changes; **c** Xenografted tumors and tumor weight; **d** RT-qPCR to detect HDAC2 expression in xenografted tumor tissues; **e** RT-qPCR to detect miR-503-5p expression in xenografted tumor tissues; **f** HE staining to observe xenografted tumor tissues; **g** Relative Ki-67 expression in xenografted tumor tissues; **h** relative PCNA expression in xenografted tumor tissues; for all quantitative results, the data were expressed as mean ± standard deviation of three independent experiments. Discrepancies among multiple groups were assessed by one-way ANOVA; * *P* < 0.05 compared with the sh-NC group

We also tested ESCC cell invasion and migration after knocking down HDAC2, and finally observed an impairment in ESCC cell migration and invasion rates (Fig. 3a). Then, to clarify whether HDAC2 regulated ESCC in vivo, the transfected Ec109 cells (sh-HDAC2 or sh-NC) were injected subcutaneously into the back of mice. It was depicted that Ec109 cells with reduced HDAC2 formed smaller tumor volume and lighter tumor weight (Fig. 3b, c). Also, we examined HDAC2 and miR-503-5p expression in mice tumors and found that silencing HDAC2 reduced HDAC2 and elevated miR-503-5p expression levels (Fig. 3d, e). HE staining and RT-qPCR were performed with mice tumor sections and exhibited that HDAC2 inhibition repressed tumor growth (Fig. 3f–h). In short, HDAC2 knockdown inhibited tumor growth.

### HDAC2 is bound to miR-503-5p promoter and inhibits miR-503-5p expression

**Fig. 4:**
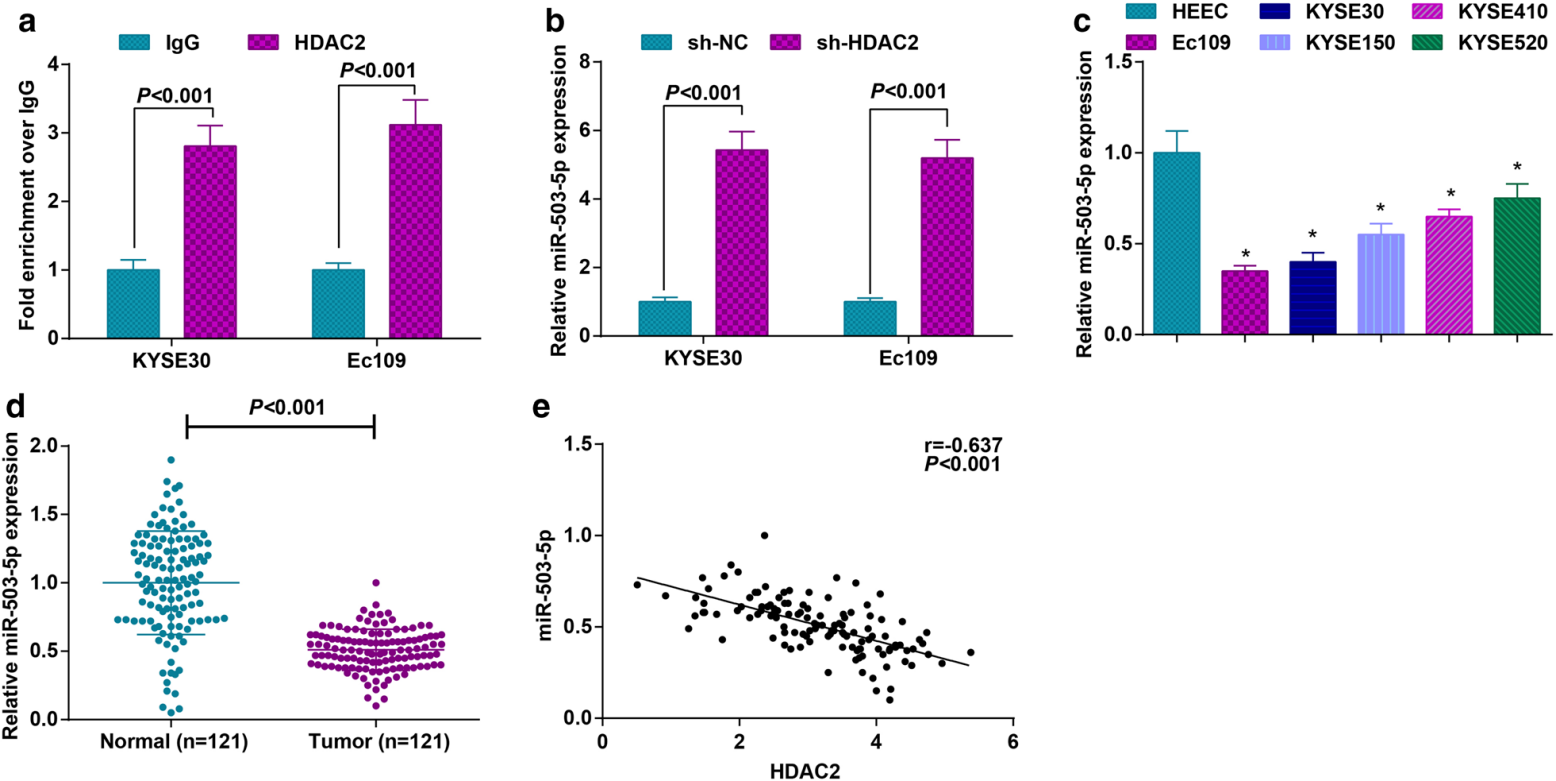
HDAC2 is bound to miR-503-5p promoter and inhibits miR-503-5p expression. a. ChIP assay to detect HDAC2 enrichment on the miR-503-5p promoter in ESCC cells; b. RT-qPCR to detect miR-503-5p expression in ESCC cells after HDAC2 knockdown; c. RT-qPCR to detect miR-503-5p mRNA expression in ESCC cell lines and normal epithelial cells; d. RT-qPCR to detect miR-503-5p expression in ESCC cancer and normal esophageal epithelial tissues; e. Pearson analysis to assess the correlation between HDAC2 and miR-503-5p in ESCC cancer tissues; for all quantitative results, the data were expressed as mean ± standard deviation of three independent experiments. Discrepancies between two groups were assessed by Student’s t test while those among multiple groups by one-way ANOVA; * *P* < 0.05 compared with HEEC cells

HDAC2 could be recruited to the miRNA promoter. For instance, in OCI-AML cells and AML cells, HDAC2 was bound to the miR-182 promoter to inhibit miR-182 expression [25]. To identify whether HDAC2 functioned in miR-503-5p in a similar manner, we carried out immunoprecipitation and detected the existence of HDAC2 through Western blot assay. Also, we recognized in ChIP assay that HDAC2 was specifically recruited to the miR-503-5p promoter in Ec109 and KYSE30 cells (Fig. 4a).

The anti-tumorigenic effect of miR-503-5p has been documented in human cancers including ESCC [11, 26]. Based on that, we measured miR-503-5p expression in Ec109 and KYSE30 cells after knocking down HDAC2 and discovered that inhibiting HDAC2 elevated miR-503-5p expression (Fig. 4b). Also, ESCC cell lines which expressed low HDAC2 were featured by up-regulated miR-503-5p (Fig. 4c). Moreover, we uncovered that miR-503-5p was lowly expressed in ESCC cancer tissues (Fig. 4d), and miR-503-5p and HDAC2 was negatively connected (Fig. 4e). The findings made it clear that HDAC2 inhibited miR-503-5p by binding to the miR-503-5p promoter.

### Restoring miR-503-5p represses ESCC cell progression

**Fig. 5:**
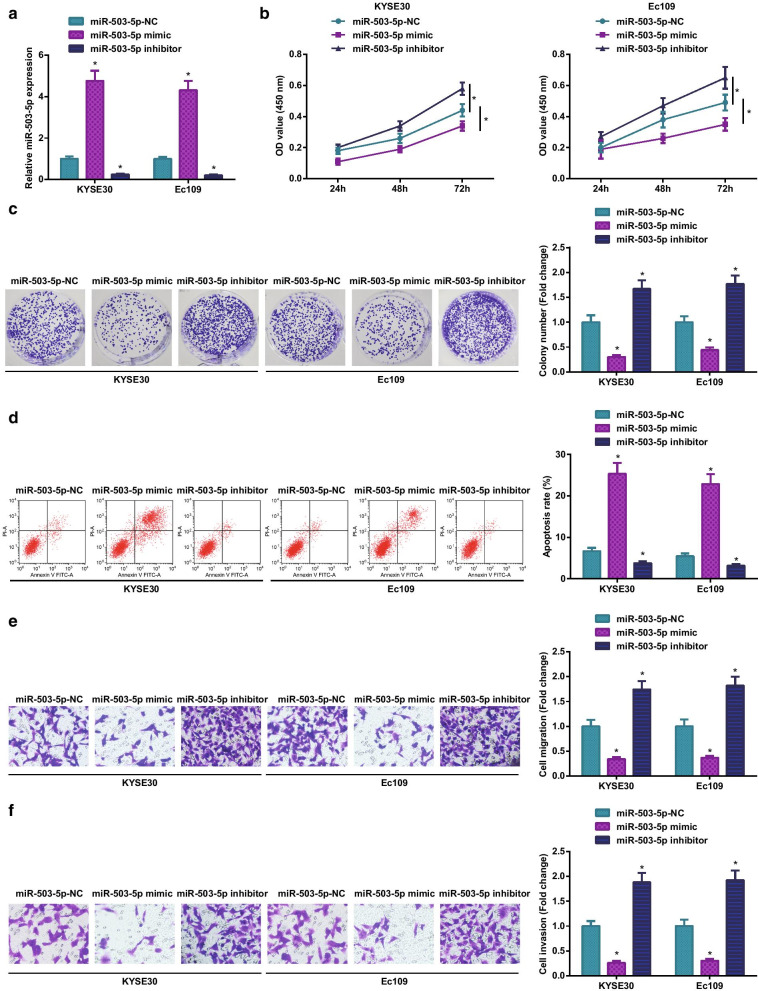
Restoring miR-503-5p represses ESCC cell progression. **a** RT-qPCR to detect miR-503-5p mRNA expression in ESCC cells; **b** MTT assay to detect ESCC cell viability; **c** colony formation assay to detect ESCC cell colony-forming ability; **d** flow cytometry to detect ESCC cell apoptosis rate; **e**–**f** transwell assay to detect ESCC cell migration and invasion abilities; for all quantitative results, the data were expressed as mean ± standard deviation of three independent experiments. Discrepancies among multiple groups were assessed by one-way ANOVA; * *P* < 0.05 compared with the miR-503-5p-NC group

For exploration of miR-503-5p involvement in ESCC, ESCC cells were transfected with miR-503-5p mimic or miR-503-5p inhibitor (Fig. 5a). After various assays, we discovered that miR-503-5p overexpression diminished viability, colony-forming, invasion and migration abilities, enhanced apoptosis of KYSE30 and Ec109 cells (Fig. 5b–f). Intriguingly, miR-503-5p knockdown functioned in an opposite way in KYSE30 and Ec109 cells.

### CXCL10 is positively connected with HDAC2 and negatively connected with miR-503-5p in ESCC

**Fig. 6:**
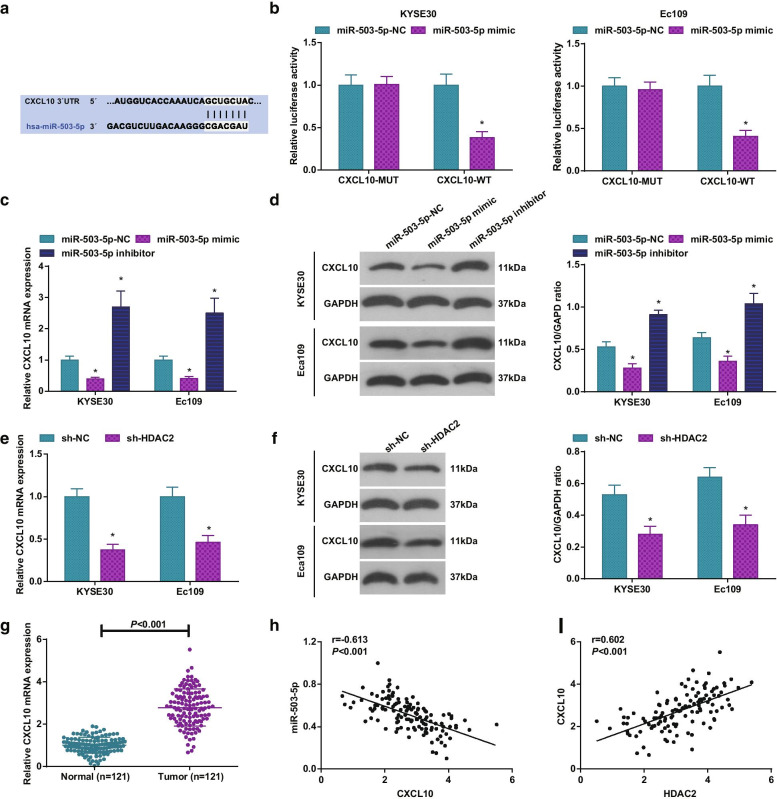
CXCL10 is positively connected with HDAC2 and negatively connected with miR-503-5p in ESCC. **a** TargetScan software to analyze the binding site of miR-503-5p and CXCL10; **b** dual-luciferase reporter gene assay to analyze the binding relation between miR-503-5p and CXCL10; **c** RT-qPCR to detect CXCL10 mRNA expression in ESCC cells after miR-503-5p expression regulation; **d** Western blot assay to detect CXCL10 protein expression in ESCC cells after miR-503-5p expression regulation; **e** RT-qPCR to detect CXCL10 mRNA expression in ESCC cells after HDAC2 expression regulation; **f** Western blot assay to detect CXCL10 expression in ESCC cells after HDAC2 expression regulation; **g** RT-qPCR to detect CXCL10 protein expression in ESCC cancer and normal esophageal epithelial tissues; **h** Pearson analysis to assess the correlation between miR-503-5p and CXCL10 in ESCC cancer tissues; **i** Pearson analysis to assess the correlation between CXCL10 and HDAC2 in ESCC cancer tissues; for all quantitative results, the data were expressed as mean ± standard deviation of three independent experiments. Discrepancies between two groups were assessed by Student’s t test while those among multiple groups by one-way ANOVA; in figure **b**–**d**, * *P* < 0.05 compared with the miR-503-5p-NC group. In figure **e**/**f**, * *P* < 0.05 compared with the sh-NC group

It was known that CXCL10 expression was elevated in clinical ESCC tissues [13]. Bioinformatics analysis revealed that miR-503-5p could bind to the 3′-untranslated region of CXCL10 (Fig. 6a) and dual-luciferase experiments detected that miR-503-5p mimic and CXCL10-WT co-transfected resulted in reduction of cell luciferase activity (Fig. 6b). Furthermore, CXCL10 expression in KYSE30 and Ec109 cells were tested after regulation of HDAC2 or miR-503-5p via RT-qPCR and Western blot assay, and the results pictured that knocking down HDAC2 or up-regulating miR-503-5p suppressed CXCL10 expression while down-regulating miR-503-5p increased CXCL10 expression (Fig. 6c–f). In clinical samples, we examined CXCL10 expression trending toward an increment (Fig. 6G) and showing a negative correlation with miR-503-5p expression (Fig. 6h) while a positive connection with HDAC2 expression (Fig. 6i).

### miR-503-5p inhibition or CXCL10 elevation negates HDAC2 knockout-induced effects on the biological functions of ESCC cells

**Fig. 7:**
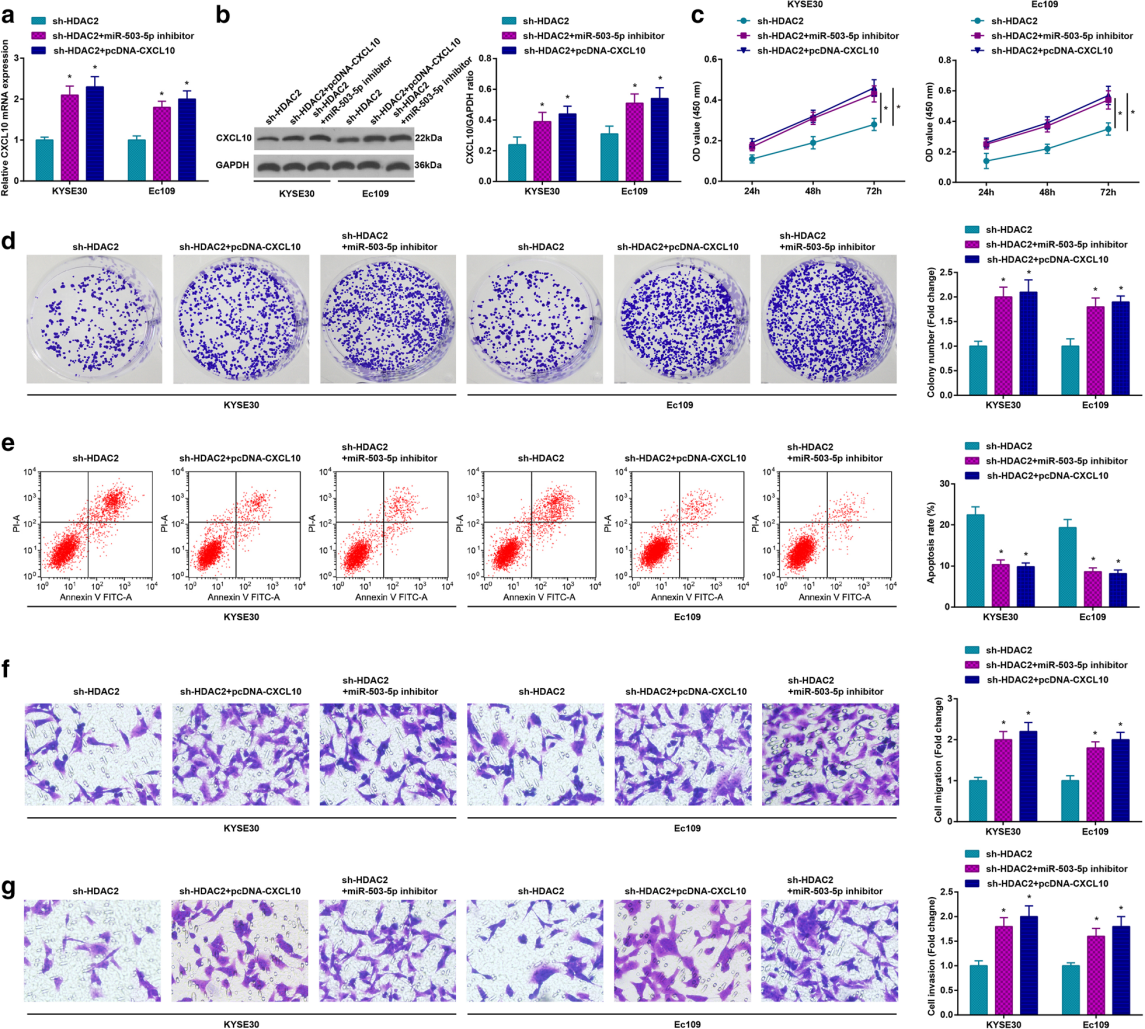
miR-503-5p inhibition or CXCL10 elevation negates HDAC2 knockout-induced effects on ESCC tumorigenesis. **a** RT-qPCR to detect CXCL10 mRNA expression in ESCC cells; **b** Western blot assay to detect CXCL10 protein expression in ESCC cells; **c** MTT assay to detect ESCC cell viability; **d** colony formation assay to detect ESCC cell colony-forming ability; **e** flow cytometry to detect ESCC cell apoptosis rate; **f** transwell assay to detect ESCC cell migration ability; **g** transwell assay to detect ESCC cell invasion ability; for all quantitative results, the data were expressed as mean ± standard deviation of three independent experiments. Discrepancies among multiple groups were assessed by one-way ANOVA;* *P* < 0.05 compared with the sh-HDAC2 group

To determine the carcinogenic effect of miR-503-5p/CXCL10-mediated HDAC2 in ESCC, KYSE30 and Ec109 cells with HDAC2 low expression were further transfected with miR-503-5p inhibitor or pcDNA-CXCL10. It was outlined that miR-503-5p inhibitor or pcDNA-CXCL10 increased CXCL10 expression (Fig. 7a) and miR-503-5p inhibitor or pcDNA-CXCL10 functionally reversed the effects of HDAC2 knockout on ESCC cells (Fig. 7b–g).

### HDAC2 inhibitor TSA inhibits the biological functions of ESCC cells

To explore the role of HDAC2 inhibitor TSA in ESCC, we conducted related experiments on KYSE30 and Ec109 cells, and treated KYSE30 and Ec109 cells with DMSO and TSA. The results displayed that after TSA treatment, cell viability, colony-forming ability, migration and invasion rates were suppressed while apoptosis was induced (Additional File 1: Fig. S1A–E). Shortly, HDAC2 inhibitor TSA can inhibit the biological functions of ESCC cells.

## Discussion

As one of the most aggressive malignancies, ESCC imposes lethal menace on human beings [27]. Illuminated by the existed knowledge about the implications of HDACs and miRNAs in ESCC, this work was started from the axis of HDAC2 and miR-503-5p with the involvement of CXCL10 in this disease. The experimental data highlighted that HDAC2 knockdown retarded ESCC progression through restoring miR-503-5p and silencing CXCL10.

At first, how HDAC2 was expressed in ESCC tissues was tested and it was manifested that HDAC2 expression was elevated in ESCC tissues which was tied up with the inferior prognosis of ESCC patients. For further elucidation of the performance of HDAC2 in ESCC, HDAC2 down-regulation assay was implemented on ESCC cells with the findings revealing that silencing HDAC2 depressed ESCC cell viability, invasion, migration and colony-forming properties, arrested cell cycle and reinforced apoptosis. Further validated by tumor xenografts in mice, depressed HDAC2 was anti-tumorigenic in ESCC. Supported by a recent research, HDAC2 expression is elevated in ESCC which is closely connected with clinical stage and lymph node metastasis, and HDACs inhibition disrupts ESCC cell progression, ameliorates cancer stemness and impedes tumorigenicity in mice [28]. In addition, another research has supplied evidence that HDAC2 is up-regulated in ESCC tissues, and HDAC2 suppression is partially attributable to ESCC cell proliferation inhibition and apoptosis reinforcement [8]. Conducted by a prior study, it is stressed out that HDAC2 expression is elevated in ESCC tissues, connecting with lymph node metastasis, invasion depth, histological grade, TNM stage and knocking down HDAC2 obstructs ESCC cells to proliferate, arrests G0/G1 phase cell cycle and accelerates apoptosis [7]. Except the aforementioned researches, there is an supplementary work demonstrating that HDAC2 is overexpressed in ESCC tissues and HDAC2 knockdown partially hinders ESCC cell invasive behaviors [29]. Anyway, these works have more or less supported the concluded results in this study.

Then, the link between HDAC2 and miR-503-5p was discovered, and the results displayed that HDAC2 was bound to miR-503-5p promoter and inhibited miR-503-5p expression. Furthermore, it was tested that miR-503-5p expression was decreased in ESCC. As to the performance of miR-503-5p in the process of ESCC, it was depicted that restoring miR-503-5p repressed ESCC cell progression. Till now, the binding link between HDAC2 and miR-503-5p has not been discussed in the existed studies, which requires to be further validated. As suggested in a study, a reduction can be seen in miR-503 expression in ESCC tissues, while miR-503 reinforcement blocks the way for ESCC cells to proliferate, invade and migrate [11]. Experimentally explored, it is recorded that miR-503-5p expression tends to decrease in the early stage of hepatocellular carcinoma [30]. Mechanistically, the decreased miR-503-5p has also been presented in cervical cancer, whereas miR-503-5p restoration blocks the way for cervical cancer cells to behave aggressively [26]. Moreover, miR-503-5p expression trends toward a decrease in ovarian cancer and its down-regulation is auxiliary for ovarian cancer cell viability while suppressive for apoptosis [31].

Next, CXCL10 was delved out to negatively connect with miR-503-5p while positively connect with HDAC2 in ESCC cells. CXCL10 expression was investigated to be overexpressed in ESCC. Moreover, another result concluded in this work was that miR-503-5p inhibition or CXCL10 elevation negated HDAC2 knockout-induced effects on ESCC tumorigenesis. Nearly no study has elucidated the regulatory connections between CXCL10 with miR-503-5p and HDAC2, which are supposed to be confirmed in the preceding explorations. Up-regulated CXCL10 has been documented in advanced thoracic ESCC tissues [13]. Similarly, overexpressed CXCL10 has been also documented in breast cancer, which can facilitate cell invasion, migration and colony formation [32, 33]. Whatever, these works focusing on CXCL10 have echoed with the concluded results in this study.

Collectively, this work has illustrated the positive performance of inhibited HDAC2 and CXCL10 and enhanced miR-503-5p in disturbing ESCC aggravation. This work has supplemented to the known mechanism of ESCC progression and renews an innovative concept for ESCC management. Bounded by the small scale of this work, a larger cohort of researches should be initiated for further confirmation and advancements.

## Supplementary Information

**Additional file 1. Fig. 1** HDAC2 inhibitor TSA inhibits the biological functions of ESCC cells. A) MTT assay to detect ESCC cell viability; B) colony formation assay to detect ESCC cell colony-forming ability; C–D) transwell assay to detect ESCC cell migration and invasion ability; E) flow cytometry to detect ESCC cell apoptosis rate; for all quantitative results, the data were expressed as mean ± standard deviation of three independent experiments. Discrepancies among multiple groups were assessed by one-way ANOVA; * *p* < 0.05 vs. the DMSO group.
